# Removable versus fixed myo-functional appliances in class II malocclusion among Indians

**DOI:** 10.6026/973206300191318

**Published:** 2023-12-31

**Authors:** Rahul Pawar, Shib Kumar Nath, P Kiran Kumar, Khyati Patel, Mengi Arvind, Alap Shah, Bela Dave, Dhaval Niranjan Mehta

**Affiliations:** 1 Department of Orthodontic and Dentofacial Orthopaedics, JCD Dental College, Sirsa, Haryana, India; 2 Consultant Orthodontist, The Smile Architect Dental Clinic and Braces Centre, Agartala, Tripura, India; 3 Department of Orthodontics and Dentofacial Orthopedics, College of Dental Sciences, Davangere, India; 4 Department of Orthodontics and Dentofacial Orthopedics, Narsinhbhai Patel Dental College and Hospital, Sakalchand Patel University, Visnagar, Gujarat, India; 5 Department of Orthodontics, Indira Gandhi Government Dental College, Jammu, India; 6 Department of Orthodontics and Dentofacial Orthopedics, Karnavati school of dentistry, Karnavati University, Uvarsad, Gandhinagar, India; 7 Department of Periodontology, Ahmedabad Municipal Corporation Dental College, Ahmedabad, Gujarat, India; 8 Department of Oral Medicine and Radiology, Narsinbhai Patel Dental college and Hospital, Sankalchand Patel University, Visnagar, Gujarat

**Keywords:** Frankal appliance, twin block, fixed orthodontic appliances, PowerScope and Forsus

## Abstract

It is of interest to compare two myofunctional appliances (frankal appliance and twin bloc) and two fixed orthodontic appliances (PowerScope and Forsus) in management of class II div 1 malocclusion. A total of 56 Class II division 1 malocclusion patients indicated for treatment with myofunctional appliances and fixed functional appliances were randomized. They were equally divided among frankal appliance (n=14), twin block appliance (n=14), PowerScope (American Orthodontics) (n=14), Forsus (3M Unitek Corp) groups (n=14). Skeletal and dentoalveolar effects of all appliances were compared. SNB increased remarkably by 4.2° in the Twin block group and it was high among all treatment groups. There was a significant decrease in vertical dimensions (SN-GoGn) in the Twin block (p = 0.002). Early treatment of Class II due to mandibular retrusion with Twin block functional appliance is recommended due to its favorable skeletal effect.

## Background:

In certain populations, class II malocclusion accounts for nearly thirty percent of orthodontic treatment cases, making it one of the most prevalent issues in orthodontic practice [1-2]. In terms of permanent dentition, Class II malocclusion had a global geographic distribution of 19.56%. It is responsible for between 12 and 49 percent of orthodontic issues [3-4. Similar to various other malocclusions, class II malocclusion results in psychological, functional and aesthetic problems. The degree of these problems is determined by the degree of antero-posterior disparity and how it interacts with the soft tissue framework around it [5,6]. Class II malocclusion is caused by a variety of reasons, the most prevalent of which is mandibular retro-gnathism [7-8]. Using growth modulation, tooth eruption and the functioning of muscles forces, various functional appliances are employed to rectify a class II malocclusion [9-10]. The effectiveness of functional appliances in promoting mandibular growth, which results in an ongoing enhancement in the skeletal pattern, is the subject of discussion on the appliances' mode of action [11-12]. Over the past few decades, the Twin Block functional appliance has emerged as the most well-liked detachable functional appliance [13-14,15]. It was the most effective in causing skeletal alterations. It may be utilised in both mixed and permanent dentition and has been shown to be aesthetic, pleasant, and effective [16-17,18]. Operator and financial considerations may influence the functional appliance selected for the treatment of Class II division 1 malocclusion [19-20]. The sole soft tissue-borne functional device that is utilised for the treatment of Class 2 Div 1 malocclusion is the Frankel Regulator 2 (FR2) appliance [2, 21-22]. One of the many fixed functional devices that orthodontists frequently utilise is the forsus fatigue resistant device (3M Unitek Corp) [18- 19]. The device consists of push rod that fits into a telescopic cylinder. It attaches to the arch wire of mandible either distal to the canine or distal to first premolar bracket. On the other hand, forsus has been linked to soft tissue injuries and canine bracket breaking frequently [19-20]. A new tool in the arsenal of orthodontists is the PowerScope (American Orthodontics, Sheboygan, Wis) [18, 20]. The appliance comes preassembled with attaching nuts for effortless chairside deployment, and it fits all sizes 8. The appliance is wire-to-wire installed, with attachments situated distal to the mandibular arch's canine and mesial to the maxillary arch's first molar [18,20]. Numerous studies have assessed the effects on the teeth and skeletal structure by the Twin Block and Frankal appliances, contrasting them other functional appliances like the Herbest applaince or the Activator appliances [21, 23,24,25]. Nevertheless, no study has examined the efficacy of fixed functional appliances, such as Forsus and PowerScope, in treating class II malocclusion in comparison to the Twin block and Frankal appliance. Therefore, it is of interest to compare two myofunctional appliances (frankal appliance and twin block) and two fixed functional appliances (PowerScope and Forsus) in management of class 2 div 1 malocclusion.

## Methods and Materials:

A total of 56 Class II division 1 malocclusion patients indicated for treatment with myofunctional appliances and fixed functional appliances were randomized and equally divided among frankal appliance (n=14), twin block appliance (n=14), PowerScope (n=14), Forsus groups (n=14). Skeletal and dentoalveolar effects of all appliances were compared. The secondary outcomes were evaluation of patient comfort and operator convenience. Cephalometric skeletal, dental, and soft tissue angular and linear measurements were used for evaluation.

## Inclusion Standards:

[1] Skeletal class II division 1 malocclusion with mandibular retrognathism was one of the inclusion criteria.

[2] Measurements of cephalometric angles: ANB ≥ 4, SNB < 78, SNA ≥ 82

[3] Overjet ≥6 mm, and

[4] A patient in stages 2 and 3 of circumpubertal development (CVM2 and CVM3).

## The following were the exclusion standards:

[1] Prior orthodontic care,

[2] Diseases of the temporomandibular joint (TMJ) or craniofacial abnormalities,

[3] Syndromes or systemic illnesses, and

[4] Oral habits are present.

After being made aware of the goal of the intervention as well as the risks and rewards involved, they signed a consent form. The patients' ages were 11 ± 1.46 years at the start of the trial.

## Interventions:

In accordance with Clark's instructions, mandibular retrognathism was corrected using a twin block device and a Frankal 2 appliance [2,13,22]. The exacto bite guided the jaw 4 mm anteriorly for the twin block appliance. If the overjet exceeded 4 mm, a second wax bite - a sequential technique of construction bite-was performed after the 4 mm overjet was corrected. To optimise the effects of all functional forces operating on the teeth, including mastication forces, the patients were directed to wear the device twenty-four hours a day for a year.

The anterior teeth of the FR2 were spaced 1-4 mm apart to make room for the lingual shield crossover wires. Participants in the FR2 group were directed to wear the appliance full time except for eating, contact sports, swimming and oral hygiene. An L-pin was used to secure the Forsus Group to the maxillary headpiece tube. For the purpose of attaching the push rod, a circular loop was positioned in the mandibular arch distal to the canine bracket. The maxillary attachment screw on the maxillary rectangular stainless steel arch wire for the PowerScope group was positioned mesially to the first molar. Using the included driver, attach the mandibular attachment to the mandibular rectangular stainless steel arch that is distal to the canine wire. Follow-up visits were planned once every 4 weeks. The antero-posterior dental arch relationship was examined, with and without the appliance, at each appointment.

## Lateral cephalometric radiographs:

Using lateral cephalogram X-ray machine (Vision X ray) lateral cephalometric radiographs was employed in the analysis. Measurements were taken of the soft tissues, teeth, and skeleton.

## Outcomes:

After a year of therapy or observation, the main results were alterations to the mandible's and maxilla's skeleton and dental structure.

## Statistical Analysis:

The statistical package for social sciences (SPSS) software was used for statistical analysis. Numerical and percentage descriptions were used for qualitative data. For regularly distributed data, continuous variables were shown as mean standard deviation (SD) Measurements taken before and after treatment were compared applying the paired t-test (also known as the Wilcoxon signed-rank test). The Student's t-test (also known as the Mann-Whitney test) was used to compare the pretreatment versus post-treatment values of the two groups. The results were deemed significant when p < 0.05.

## Results:

It was observed that there was significant increase in SNA in all the appliances (Twin block, frankal, Forsus and Powerscope) after treatment. However, the increase in SNA was significantly greater in Twin block appliance and Frankal 2 appliance as compared to fixed functional appliances (Forsus and Powerscope). When there was comparison between Twin block and Frankal 2 appliance then the difference between them was not statistically meaningful. Similarly, on comparing Forsus to Powerscope there was no statistically meaningful difference between them. The SNA values before treatment and after treatment was 82.17 ± 4.37° and 81.91 ± 3.72° in Twin block. The SNA values before treatment and after treatment was 80.05± 3.20° and 79.89 ± 2.61° in Frankal appliance. The SNA values before treatment and after treatment was 82.86 ± 2.50 ° and 82.03 ± 3.93° in Forsus. The SNA values before treatment and after treatment was 82.86 ± 2.50° and 82.03 ± 3.93° in powerscope (Table 1). On analyzing SND values, it was found that there was significant increase in its values after treatment in Twin block and Frankal appliance. However there was no significant increase observed in case of Forcus and Powerscope fixed functional appliance. It was observed that there was significant increase in its values of N-A-Pog after treatment in Twin block and Frankal appliance. However there was no significant increase observed in case of Forcus and Powerscope fixed functional appliance. It was observed that there was significant decrease in ANB in all the appliances (Twin block, frankal, Forsus and Powerscope) after treatment. However, the decrease in ANB was significantly greater in Twin block appliance and Frankal 2 appliance as compared to fixed functional appliances (Forsus and Powerscope). When there was comparison between Twin block and Frankal 2 appliance then the difference between them was not statistically meaningful. Similarly, on comparing Forsus to Powerscope there was no statistically meaningful difference between them (Table 2). On analyzing vertical relationship it was observed that values of SN-GoGn decreased significantly (p=0.008) in Twin block and frankal appliance (p=0.005). However no significant change was observed in values of of SN-GoGn in Forsus and Powerscope. It was observed that values of FMA decreased significantly (p=0.035) in Twin block and frankal appliance (p=0.013). However no significant change was observed in values of SN-GoGn in Forsus and Powerscope. In all other parameters (AFH, UAFH, LAFH, PFH) there was significant changes in all appliances studied (Table 2). When there was analysis of cranial base measurements then there was significant decrease in values of N-S-Ar in twin block ( p=0.007) and Frankal appliance (0.006). However there was no significant change in Forsus and Powerscope appliances. Rest of parameters in cranial base measurements (S-Ar-Go, N-Se , S-Ar ) showed significant variation in results post treatment in all appliances. However, the overall improvement in cranial base measurements was better in twin block and Frankal appliances (Table 3). When there was analysis of mandibular measurements there was significant changes in all parameters evaluated (Ar-Go-Me, Co-Go, Co-Gn, Go-Gn, Go-Pog, Go-Me) in all appliances. However there was slight more increase in Ar-Go-Me in Twin block and Frankal appliance (Table 4).

On analysis of dental parameters and soft tissue parameters it was observed that there was significant changes in all parameters except L6-MP in both Twin block and Frankal appliance. On the other hand it was observed that there were significant changes in all parameters except L1-MP, Overjet, overbite in Forcus and Powerscope. There was siginificant reduction in overjet and overbite in Frankal and Twin block appliance. Although there was reduction in overjet and overbite in Forcus and Poerscope , but the variation was non-significant statistically. In the Frankal group and Twin block group, SNB rose noticeably by 4.1°, but in Forcus and Powerscope group, it increased by just 0.67. The Twin block and Frankal group's decrease in vertical dimensions (SN-GoGn) were significant than those of the Forcus and Powerscope group. There was a noticeable improvement in the patients' facial profiles in all appliances, but the results were much better in Twin block and Frankal appliance.

## Discussion:

Numerous studies have assessed the effects on the teeth and skeletal structure by the Twin Block and Frankal appliances [12-16]. Nevertheless, no study has examined the efficacy of fixed functional appliances, such as Forsus and PowerScope, in treating class II malocclusion in comparison to the Twin block and Frankal appliance. This study was carried out to compare two myofunctional appliances (frankal appliance and twin block) and two fixed functional appliances (PowerScope and Forsus) in management of class 2 div 1 malocclusion. Data shows that there is more improvement in SNA angle in Twin block appliance [2,16,17, 22]. They also showed that improvement in anteroposterior relation is better in case of Twin block and Frankal appliance [2,16,18, 22]. The Forsus fatigue resistant device is one of the numerous fixed functional devices that orthodontists commonly use. A push rod that slides into a telescopic cylinder makes up the gadget. Either distal to the canine or distal to the first premolar bracket, it is attached to the mandibular arch wire. Conversely, Forsus has been connected to frequent canine bracket breaking and soft tissue injuries [19].The PowerScope is a new instrument in the toolbox of orthodontists. The appliance fits all sizes and comes preassembled with mounting bolts for simple chairside deployment. The appliance is wire-to-wire installed, with attachments mesial to the first molar of the maxillary arch and distal to the canine of the mandibular arch [18-19]. There was observations that decrease in vertical dimensions was greater in Twin block appliances. There was also decrease in vertical dimensions in frankal appliances [20-25]. There are several causes of class II malocclusion, the most common being mandibular retrognathism. To treat a class II malocclusion, a variety of functional appliances are used in conjunction with muscle forces, growth regulation, and tooth eruption [4]. We talk about the mechanism of action of functional appliances and how well they promote mandibular growth, leading to a continuous improvement in the skeletal pattern [5-10]. The Twin Block functional appliance has become the most popular detachable functional appliance during the last few decades. Additionally, systematic evaluations have demonstrated that it was the most successful in altering the skeleton [16]. It has been demonstrated to be aesthetically pleasing, pleasurable to use, and effective in both mixed and permanent dentition.

When treating Class II division 1 malocclusion, the functional appliance chosen may be influenced by operator and budgetary factors. The Frankel regulator 2 (FR2) appliances is the only soft tissue-borne functional device used to treat Class 2 Div 1 malocclusion [22-24]. Several studies have shown the similar significant decrease in values of parameters of cranial measurements in Twin block appliances [5- 10]. Class II malocclusion is one of the most common problems in orthodontic therapy, accounting for approximately thirty percent of cases requiring orthodontic treatment in some populations [10]. Class II malocclusion exhibited a global geographic distribution of 19.56% with regard to permanent dentition. It accounts for twelve to forty-nine percent of orthodontic problems [21]. Class II malocclusion causes psychological, functional and cosmetic issues, just like a number of other malocclusions. The degree of antero-posterior discrepancy and its interaction with the surrounding soft tissue framework determine the severity of these issues.

## Limitations:

The brief duration of the investigation limits the ability to make firm conclusions. As a result, a lengthy follow-up with the patients involved in the current trial is scheduled.

## Conclusion:

Twin block appliance and Frankal appliance is preferable to fixed functional appliance in severe class II malocclusion in initial phase with increased over jet because they show a significant and remarkable advancement of the mandible.

## Figures and Tables

**Table 1:** Antero-posterior relationship

		**SNA (°)**	**SNB (°)**	**SND (°)**	**N-A-Pog (°)**	**Co-A (°)**	**ANB (°)**
Twin block	T0	82.17 ± 4.37	75.66 ± 4.20	72.35 ± 4.17	16.61 ± 6.43	104.15 ± 12.87	7.62 ± 3.24
	T1	81.91 ± 3.72	79.66 ± 3.85	75.32 ± 4.44	13.92 ± 6.76	109.62 ± 11.68	3.45 ± 2.41
P value		0.798	≤0.001*	≤0.001*	≤0.001*	≤0.001*	≤0.001*
Frankal	T0	80.05± 3.20	73.66 ± 4.20	70.13 ± 3.05	14.40 ± 4.21	102.02 ± 10.65	5.41 ± 2.04
	T1	79.89 ± 2.61	75.44 ± 2.62	73.10 ± 2.22	12.81 ± 5.65	107.40 ± 09.46	2.34 ± 1.21
P value		0.465	≤0.001*	≤0.001*	≤0.001*	≤0.001*	≤0.001*
Forsus	T0	82.86 ± 2.50	76.13 ± 2.14	73.18 ± 3.57	16.16± 4.04	107.89 ± 15.53	6.73 ± 1.74
	T1	82.03 ± 3.93	76.81 ± 2.56	71.88 ± 3.00	15.55 ± 3.72	110.15 ± 15.48	6.32 ± 1.85
P value		0.602	0.01*	0.835	0.45	≤0.001*	0.019*
PowerScope	T0	80.64 ± 1.49	74.01 ± 2.03	71.06 ± 1.35	15.05 ± 3.93	105.78 ± 14.42	5.62 ± 1.63
	T1	81.92 ± 2.82	74.69 ± 2.34	70.77 ± 3.00	15.55 ± 3.72	108.04 ± 14.37	6.11 ± 1.62
P value		0.591	0.01*	0.724	0.349	≤0.001*	0.018*

**Table 2:** Vertical relationship

		**SN-GoGn (°)**	**FMA (°)**	**AFH (mm)**	**UAFH (mm)**	**LAFH (mm)**	**PFH (mm)**
Twin block	T0	36.59 ± 6.77	24.90 ± 6.69	138.13 ± 7.46	62.43 ± 6.13	79.26 ± 3.74	84.00 ± 9.88
	T1	35.15 ± 7.53	23.65 ± 6.79	142.30 ± 8.12	64.96 ± 6.59	82.52 ± 5.33	87.93 ± 9.59
P value		0.008*	0.035*	≤0.001*	≤0.001*	≤0.001*	≤0.001*
Frankal	T0	35.48 ± 5.66	23.89 ± 5.58	137.02 ± 6.35	61.32 ± 5.02	78.15 ± 2.63	83.90 ± 8.77
	T1	34.04 ± 6.42	22.54 ± 5.68	141.29 ± 7.01	63.85 ± 5.48	81.51 ± 4.22	86.82 ± 9.48
P value		0.005*	0.013*	≤0.001*	≤0.001*	≤0.001*	≤0.001*
Forsus	T0	34.47 ± 7.90	23.36 ± 4.73	137.62 ± 9.60	60.37 ± 7.28	77.82 ± 4.56	84.53 ± 11.20
	T1	36.15 ± 6.62	23.67 ± 4.79	141.87 ± 8.79	66.20 ± 8.13	83.68 ± 5.82	90.50 ± 11.58
P value		0.325	0.23	≤0.001*	≤0.001*	≤0.001*	≤0.001*
Power Scope	T0	36.69 ± 6.79	25.58 ± 4.95	139.73 ± 9.60	62.48 ± 7.28	78.82 ± 4.56	85.53 ± 11.20
	T1	37.25 ± 6.52	25.97 ± 4.79	143.97 ± 9.79	68.20 ± 8.33	85.89 ± 6.83	92.51 ± 12.59
P value		0.103	0.23	≤0.001*	≤0.001*	≤0.001*	≤0.001*

**Table 3:** Cranial base measurements

		**N-S-Ar (°)**	**S-Ar-Go (°)**	**N-Se (mm)**	**S-Ar (mm)**
Twin block	T0	130.77 ± 7.40	139.93 ± 10.67	82.58 ± 9.27	40.92 ± 5.86
	T1	127.38 ± 6.37	142.02 ± 10.43	84.69 ± 8.72	41.05 ± 5.93
P value		0.007*	≤0.001*	≤0.001*	≤0.001*
Frankal	T0	128.55 ± 5.28	137.71 ± 8.45	80.25 ± 6.03	37.60 ± 3.64
	T1	125.16 ± 4.15	140.80 ± 8.21	82.47 ± 6.50	40.94 ± 3.72
P value		0.006*	≤0.001*	≤0.001*	≤0.001*
Forsus	T0	127.70 ± 6.23	145.56 ± 6.22	82.75 ± 10.81	40.79 ± 6.98
	T1	129.63 ± 5.26	146.47 ± 5.09	86.01 ± 9.67	44.10 ± 5.86
P value		0.053	0.007*	0.002*	≤0.001*
Power Scope	T0	125.60 ± 4.01	143.56 ± 4.00	80.75 ± 8.81	38.79 ± 4.98
	T1	127.53 ± 5.26	144.47 ± 3.09	84.01 ± 7.67	42.10 ± 3.86
P value		0.053	0.007*	0.002*	≤0.001*

**Table 4:** Mandibular measurements:

		**Ar-Go-Me (°**)	**Co-Go (mm)**	**Co-Gn (mm)**	**Go-Gn (mm)**	**Go-Pog (mm)**	**Go-Me (mm)**
Twin block	T0	125.39 ± 7.76	65.10 ± 8.29	128.70 ± 15.31	87.86 ± 6.89	88.20 ± 8.57	86.96 ± 8.11
	T1	127.30 ± 8.30	68.43 ± 7.3	136.57 ± 13.95	91.85 ± 8.40	92.88 ± 9.32	92.39 ± 9.43
P value		0.002*	≤0.001*	0.001*	≤0.001*	≤0.001*	≤0.001*
Frankal	T0	123.17 ± 6.65	63.08 ± 6.28	126.69 ± 14.21	85.76 ± 5.78	86.20± 8.56	84.95 ± 7.20
	T1	123.07 ± 7.29	65.43 ± 6.33	133.46 ± 12.74	88.63 ± 7.39	90.77 ± 8.21	90.28 ± 8.32
P value		0.003*	≤0.001*	0.001*	≤0.001*	≤0.001*	≤0.001*
Forsus	T0	126.45± 6.09	66.73 ± 9.12	132.67 ± 15.23	87.90 ± 12.25	88.23 ± 13.21	85.75 ± 11.98
	T1	127.74 ± 6.28	71.82 ± 12.35	140.57 ± 17.05	93.58 ± 13.06	94.98 ± 13.58	94.80 ± 12.40
P value		0.008*	≤0.001*	≤0.001*	≤0.001*	≤0.001*	≤0.001*
Power Scope	T0	127.13 ± 4.87	66.51 ± 4.91	132.47 ± 14.12	87.78 ± 11.14	94.01 ± 12.10	94.64 ± 10.87
	T1	127.61 ± 6.19	71.70 ± 11.13	140.57 ± 17.03	87.58 ± 13.03	94.98 ± 13.48	94.79 ± 12.30
P value		0.008*	≤0.001*	≤0.001*	≤0.001*	≤0.001*	≤0.001*
